# Intermittent presumptive treatment in pregnancy with sulfadoxine–pyrimethamine: a counter perspective

**DOI:** 10.1186/s12936-015-0765-5

**Published:** 2015-06-20

**Authors:** Francois Nosten, Rose McGready

**Affiliations:** Nuffield Department of Medicine, Centre for Tropical Medicine, University of Oxford, Oxford, UK; Shoklo Malaria Research Unit, Mahidol-Oxford Tropical Medicine Research Unit, Faculty of Tropical Medicine, Mahidol University, Mae Sot, Thailand

## Abstract

Malaria continues to cause devastation during pregnancy. Unfortunately, there is still no clear strategy to effectively protect pregnant women and countless mothers living in malaria endemic countries are dying every year. The effective prevention of malaria during pregnancy will take much more than the so-called “Global Call for Action” for an intervention (IPTp-SP) that cannot succeed. A new and truly “global” strategy is urgently needed.

## Background

Despite recent progress, malaria continues to cause devastation during pregnancy. Not so long ago, asymptomatic pregnant women infected with *Plasmodium falciparum* in Africa were left untreated. However it is now clear that even when silent, a low-density parasitaemia is deleterious to the mother and the fetus [1]. In areas of intense transmission, *P. falciparum* kills the mothers because they become severely anaemic, while in area of low transmission, they die of severe and cerebral malaria. *Plasmodium vivax* is also responsible for maternal anaemia, poor fetal growth and the loss of the fetus in early pregnancy [2]. All this suffering affecting poor rural communities is readily preventable: malaria parasites already present in the maternal circulation must be promptly detected and eliminated and new infections totally prevented. Unfortunately, there is no clear strategy to achieve this objective and countless mothers are dying every year. A new and truly “Global” strategy is urgently needed.

In their opinion paper, Chico and colleagues [3] lament at the low up-take of intermittent preventive treatment with sulfadoxine–pyrimethamine (SP) (IPTp-SP) in pregnant women at risk of malaria. They call upon all “key stakeholders” nationally and internationally to join their “Global Call to Action for the scale-up of IPTp”. A detailed list of actions is proposed that will, in their view, lead to achieve rapidly maximum coverage and public health impact. However, this rather desperate “plea” is unlikely to be heard, for several reasons:

1. The call is not “global” since it focuses only on certain areas of sub-Saharan Africa. In areas of low transmission, the malaria attributable maternal mortality can be higher than that in hyper-endemic Africa [4]. Chico et al. start their paper with the usual misleading statement, about *preventing* malaria in pregnancy, while in fact IPTp-SP (sometimes and more correctly referred to as Intermittent *Presumptive* treatment [5]) is about episodic treatments of *P. falciparum* infections in pregnant women living in rapidly shrinking areas of Africa where SP remains effective, *de*-*facto* ignoring the millions of women exposed to all malaria species elsewhere in Africa and in the rest of the world.
2. IPTp-SP is essentially taking advantage of the post-treatment prophylactic effect of the slowly eliminated pyrimethamine [5] so it amounts to intermittent prophylaxis, leaving the women unprotected for significant period of time. Sulfadoxine–pyrimethamine is an old anti-malarial used in the treatment of uncomplicated *P. falciparum* infections. Its main advantages are that it can be used as a single dose and that its slow elimination provides a period of protection against new infections for several weeks after treatment. It is not used at prophylactic doses because of the risk of serious toxicity. Resistance to SP emerged in South East Asia in 1970 and has spread to large parts of Africa, where ACT has replaced it. Given its pharmacokinetic properties, SP in four treatment doses cannot truly “prevent” malaria in pregnant women for the duration of the gestation. It can only temporarily eliminate and suppress parasitaemia in areas where *P. falciparum* is still susceptible. But new infections will occur once the drug is eliminated. That’s far from the concept of prevention.
3. SP is a failed drug in large parts of Africa and the more recent studies show that the often quoted positive but indirect impacts (on birthweight and anaemia) are seriously compromised by resistance [1, 6]. It can also cause harm by increasing placental proliferation of resistant parasites [7], and by increasing gametocyte carriage. There is no direct evidence to support the claim by Chico et al. [3] that IPTp-SP reduces maternal mortality. It also unclear whether the dose administered to pregnant women is adequate. In any case, the call to action comes far too late for SP and a complete change of action is required [8].
4. The evidence for the beneficial effects of IPTp-SP is weak. The preferred (because easier to measure) endpoints in IPTp trials are maternal anaemia and birthweight. However, these are indirect markers of malaria infection and surely the absence of parasites in the maternal circulation (including the placenta) during gestation should be the primary objective and maternal mortality the primary endpoint. There is ample evidence that even asymptomatic low parasitaemia at any time during pregnancy has deleterious effects on the mother and the fetus. If one can really *prevent* malaria parasites to infect the mother during pregnancy, then all their deleterious impacts will be prevented as well. In poor, malaria affected communities in the rural world, women do not really care whether their baby is born 100 g heavier or not. They have no time for this. They are often more preoccupied with the daily struggle for survival. Call for action should be about interventions that increase their chance of survival. IPTp-SP at each routine ANC visit will not do this, because it cannot.
5. One puzzling question is that of double standards in WHO recommendations: for the treatment of malaria it recommends that new anti-malarial drugs should have >95% parasitological efficacy and that they should be replaced if the efficacy drops below 90%. So why does WHO continue to recommend using drugs in pregnancy that have much lower rates of efficacy against a potential fatal infection?

The effective prevention of malaria during pregnancy will take much more than a “Global Call for Action” for a strategy (IPTp-SP) that cannot succeed. Mothers are dying even in areas of low and unstable transmission, and fetuses are being lost to malaria even when the mothers have no symptoms. So unless the protection against the malaria parasite is effective in the first place, the goal will remain elusive. A substantial reduction of maternal mortality and morbidity will probably result from a combination of interventions: the overall decline of malaria transmission in the entire population, the protection of the mothers with LLINs and other vector control measures, and the intelligent use of safe anti-malarials that can kill any existing parasites in the circulation and prevent any new ones from infecting the women.

As experience has shown for the treatment of severe malaria [9], only strong and irrefutable evidence will change policy. What one should be calling for is a large international mortality trial comparing IPTp-SP with an effective drug regimen, for example monthly DHA–piperaquine [10], including detailed pharmacokinetic assessments to ensure correct dosing.

## References

[1] Cottrell G, Moussiliou A, Luty AJ, Cot M, Fievet N, Massougbodji A (2015). Submicroscopic *Plasmodium falciparum* infections are associated with maternal anemia, premature births, and low birth weight. Clin Infect Dis.

[2] McGready R, Lee S, Wiladphaingern J, Ashley E, Rijken M, Boel M (2012). Adverse effects of falciparum and vivax malaria and the safety of antimalarial treatment in early pregnancy: a population-based study. Lancet Infect Dis.

[3] Chico RM, Dellicour S, Roman E, Mangiaterra V, Coleman J, Menendez C (2015). Global call to action: maximize the public health impact of intermittent preventive treatment of malaria in pregnancy in sub-Saharan Africa. Malar J.

[4] Rijken MJ, McGready R, Boel ME, Poespoprodjo R, Singh N, Syafruddin D (2012). Malaria in pregnancy in the Asia-Pacific region. Lancet Infect Dis.

[5] White NJ (2005). Intermittent presumptive treatment for malaria. PLoS Med.

[6] McGready R, White NJ, Nosten F (2011). Parasitological efficacy of antimalarials in the treatment and prevention of falciparum malaria in pregnancy 1998 to 2009: a systematic review. BJOG.

[7] Harrington WE, Mutabingwa TK, Kabyemela E, Fried M, Duffy PE (2011). Intermittent treatment to prevent pregnancy malaria does not confer benefit in an area of widespread drug resistance. Clin Infect Dis.

[8] Harrington W, McGready R, Muehlenbachs A, Fried M, Nosten F, Duffy P (2012). Intermittent preventive treatment in pregnancy with sulfadoxine–pyrimethamine: the times they are a-changin’. Clin Infect Dis.

[9] Treatment Guidelines for the Treatment of Malaria (2015). http://www.who.int/malaria/publications/atoz/9789241549127/en/. Accessed 5 June 2015

[10] Lwin KM, Phyo AP, Tarning J, Hanpithakpong W, Ashley EA, Lee SJ (2012). A randomised, double-blind, placebo controlled trial of monthly versus second monthly dihydroartemisinin–piperaquine chemoprevention in adults at high risk of malaria. Antimicrob Agents Chemother.

